# Resumption of the treatment of non‐COVID‐19 gynecologic patients after lifting lockdown: Triage and infection prevention experiences from Wuhan

**DOI:** 10.1111/jog.14917

**Published:** 2021-07-28

**Authors:** Weihong Dong, Rui Gao, Jing Cai, Shouhua Yang, Jianfeng Guo, Jing Zhao, Zehua Wang, Liqiong Cai

**Affiliations:** ^1^ Department of Obstetrics and Gynecology Union Hospital, Tongji Medical College, Huazhong University of Science and Technology Wuhan China

**Keywords:** buffering room, gynecologic surgery, pandemic, personal protective equipment, triage strategy

## Abstract

**Aim:**

To share our experiences of resuming the treatments for gynecologic patients after lifting the lockdown in a hotspot area for the Coronavirus Disease 2019 (COVID‐19) pandemic.

**Methods:**

The triage process used to resume medical activities for gynecologic patients at the Wuhan Union Hospital after a 76‐day lockdown of the city is described, and its effectiveness in preventing COVID‐19 nosocomial transmission is shown.

**Results:**

Nonemergency patients were pretriaged based on their contact history and body temperature at an outpatient clinic, and negative COVID‐19 screening test results were required for admission to the buffering rooms at the gynecologic department. The buffering lasted at least 3 days for symptom monitoring, and a second round of COVID‐19 testing was required before patients could be transferred to the regular gynecologic wards. For patients who needed emergency surgery, the first screening was completed at the isolation wards after surgery, followed by buffering at the gynecologic department. We received 19 298 outpatient visits, admitted 326 patients, and performed 223 operations in the first 2 months after the lockdown was lifted. No COVID‐19 cases occurred in the hospitalized patients, while the proportion of potentially high‐risk patients with cancer and severe anemia was increased in comparison to that observed during the same period in 2019 and the first 2 months of 2020 before the lockdown.

**Conclusions:**

We provide an effective triage system with buffering at two levels to guarantee safe and timely treatment for non‐COVID‐19 gynecologic patients in the postlockdown phase.

## Introduction

Since the end of 2019, the Coronavirus Disease 2019 (COVID‐19) pandemic caused by severe acute respiratory syndrome coronavirus 2 (SARS‐CoV‐2) infection has been spreading rapidly, affecting over 2 hundred countries around the world. The novel coronavirus is highly contagious, and effective antiviral drugs are lacking. Vaccines against COVID‐19, which are considered the most promising measure to contain the pandemic.^1^ Lockdown is an effective public health measure to eliminate the coronavirus infection or flatten the outbreak curve,^2^ and many countries significantly affected by the pandemic have issued stay‐at‐home orders and requested self‐restraint to ensure reduced social interaction.^3^, ^4^ Unfortunately, a long‐term lockdown could be associated with severe social problems, particularly economic recession. In some places, the lockdowns have been gradually lifted, and social activities are slowly resuming. However, the pandemic still continues, and the lockdowns will have lasting impacts on clinical practice.

Social distancing, hand hygiene, and face mask are recommended to avoid virus transmission between patients and medical staffs during treatment of gynecologic patients. However, close proximity of patients to the gynecologist, particularly the direct contact with vaginal mucosa and secretions of the patient in a gynecologic examination, is inevitable and increases the risk of SARS‐CoV‐2 transmission. Several published guidelines have recommended safe gynecologic practices and treatment for gynecologic patients during the ongoing pandemic, especially focusing on the management of cancer patients.^5^, ^6^, ^7^ When the outbreak peak is gone and the lockdown lifted, medical resources should be redirected to non‐COVID‐19 patients, while the threat of COVID‐19 transmission still exists. In this context, an adapted triage strategy plays a central role.

Wuhan, China, the first epicenter of the COVID‐19 pandemic, was completely shut down between January 23, 2020 and April 8, 2020. According to statistics from Wuhan Health Committee, from April 1 to April 8, 2020, there was one new confirmed case and less than 200 asymptomatic infected cases of COVID‐19 in Wuhan. With the effective control of COVID‐19 in Wuhan, the treatment of non‐COVID‐19 patients has gradually become the focus of medical staff. As one of the largest medical centers in central south China, Wuhan Union Hospital, Tongji Medical College, Huazhong University of Science and Technology was assigned to treat non‐COVID‐19 patients beginning February 26, 2020. Here, we share our triage process supporting the resumption of medical activities in a gynecologic department of a comprehensive hospital and outline the scenarios emerging in our gynecologic wards in the transitional period from lockdown to reopening of the city. This may offer important lessons for colleagues who struggle to restart work after a COVID‐19 lockdown.

## Methods

The triage process used to triage non‐COVID‐19 gynecologic patients at the Department of Obstetrics and Gynecology, Wuhan Union Hospital, after a 76‐day lockdown of the city is described. This triage system was established based on considerations regarding the availability of personal protective equipment, the capacity for COVID‐19 testing, and the experience and lessons learned in the treatment of COVID‐19 patients in the Cancer Center and the West Campus of Wuhan Union Hospital, which functioned as a designated institution for the admission of COVID‐19 patients during the lockdown. The reinforcement medical teams, which were mainly consisted of specialists in serious infections and management of respiratory tract diseases, discussed and approved our proposal.^8^ To investigate its effectiveness in preventing COVID‐19 nosocomial transmission, the resumption performance in the first 2 months immediately after reopening (April and May 2020) was summarized. In addition, the characteristics of the inpatients in the gynecologic department running under the triage framework in April and May 2020 were retrospectively compared with the same period last year (April and May 2019) and the last 2 months before the lockdown (October and November 2020). The study was approved by the Ethical Review Committee of the Union Hospital, Tongji Medical College, Huazhong University of Science and Technology, ethics board approval number is 20210109.

## Results

### Personal protection of medical staff

It is mandatory for all medical staff to receive a COVID‐19 triple‐test screening before returning to work and report personal health status regarding fever and respiratory symptoms daily via a mobile phone app. Additionally, to inform possible close contacts timely, a computer program was designed to automatically capture the positive results of COVID‐19 tests and send messages to staffs who had potential exposure. In the presence of any suspicious symptoms or exposure to the virus without adequate medical personal protection, the medical staff had to stopping work and were subjected to screening tests. They were further required to undergo a 14‐day medical surveillance for daily body temperature and respiratory symptom monitoring and repeated nucleic acid and serum antibody test.

The use of PPE is summarized in Table 1. Briefly, level I PPE is recommended for low risk of exposure (gynecologic normal wards) and level II PPE for moderate and high risk of exposure (outpatient department and gynecologic buffering rooms). The medical staff who had had contact with individuals confirmed or suspected to have COVID‐19 during medical care work with adequate medical defense were considered nonexposed, and no management was required.

**TABLE 1:** Personal protective equipment for medical staff in the gynecologic department of Wuhan Union Hospital after lifting the lockdown in Wuhan

Zones	Exposure risk	Defense measures	Defense class
Normal wards	Low risk	Standard gown Disposable surgical cap Surgical mask	I
Normal outpatient and gynecologic buffering wards	Moderate risk	Protective suite^a^ Disposable surgical cap Medical protective mask Protective goggles Disposable gloves	II
Gynecologic emergency	High risk	Protective suite^a^ or protective cover^b^ Disposable surgical cap Medical protective mask Protective goggles Disposable gloves	II

^a^ AAMI PB70 level 1–3.

^b^ AAMI PB70 level 4.

### Triage and treatment of nonemergency patients

In the resumption period, all nonemergency patients underwent screening for COVID‐19 before being admitted. Before patients saw their doctors in the outpatient clinic, they were primarily triaged according to symptoms (including fever, cough, dysosmia, dysgeusia, sore throat, and nasal congestion) and their epidemiologic history of COVID‐19 by nurses with defense level III PPE. Asymptomatic women with no contact history were allowed to visit doctors under defense level II PPE. If patients needed ambulatory surgery or hospitalization, they were subjected to first COVID‐19 screening tests (nucleic acid and antibodies tests to SARS‐CoV‐2) combined with chest computerized tomography (CT) scans, which take 4 to 6 h. Only patients without any positive results could be admitted, while those with confirmed or suspected COVID‐19 were sent to designated outpatient clinics.

**FIGURE 1:**
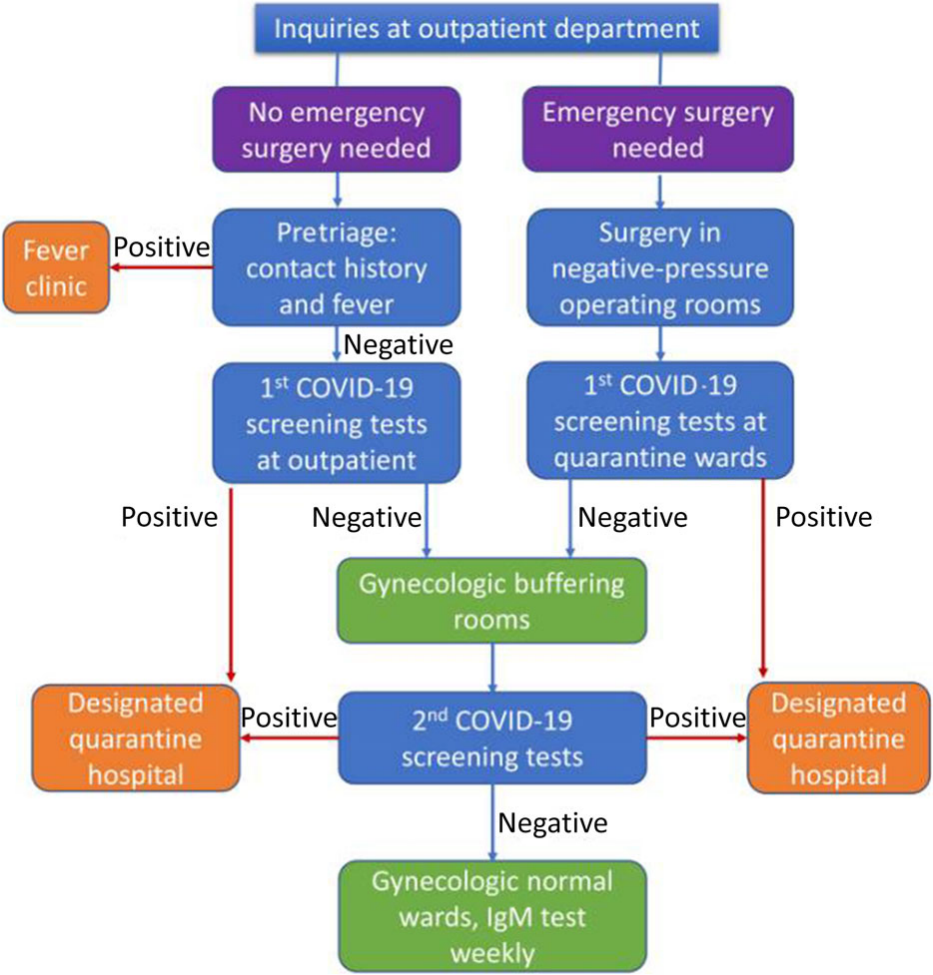
The triage process used to triage non‐COVID‐19 gynecologic patients at the Department of Obstetrics and Gynecology

The gynecologic wards are under closed management, banning visits. After admission, patients first lived in the buffering rooms for at least 3 days, which are single rooms physically separated from normal wards, and were constructed based on the principle of three zones (the clean, semi‐clean, and contaminated zones) and two channels (the separate patient and medical staff passages).^8^, ^9^ During the buffering, a second COVID‐19 screening tests (nucleic acid and antibodies tests to SARS‐CoV‐2) were performed with a time interval of more than 24 h to the previous test. Thereafter, if no positive finding was present, patients were transferred to normal wards to receive further disease‐related treatment. During hospitalization, the respiratory symptoms and body temperature were monitored daily, the specific antibodies to SARS‐CoV‐2 test was repeated weekly, if emerging fever (≥37.3°C for 3 days or longer), or any respiratory syndromes were indications for a quarantine‐in‐place and a repeated screening program (Figure 1).

With regard to elective surgery, a higher priority was given to patients with malignant tumors or benign diseases that significantly affect quality of life, for example, uterine submucous leiomyomas causing severe anemia or bulky pelvic tumors pressing the bladder or rectum. Although there have been recommendations on gynecological surgery and chemotherapy amid the COVID‐19 pandemic and debate about the COVID‐19‐related safety of open versus minimally invasive surgery,^7^, ^8^, ^10^ we did not change our surgery modalities or chemotherapy regimens because we have sufficient medical resources to provide standard care for patients, and the evidence of the risk of SARS‐CoV‐2 transmission associated with surgery modalities is lacking. Given that postponing chemotherapy may increase the risk of cancer progression, chemotherapies were administered as scheduled if possible. Moreover, adverse effects of chemotherapy, such as myelosuppression and hepatic dysfunction, were prevented as much as possible to decrease the possibility of additional hospital visits for the diagnosis and treatment of such severe side effects.

### Triage and treatment of emergency patients

For emergency gynecologic patients, whose situation allowed a surgical treatment with a waiting time of 4 h or longer, a COVID‐19 triple‐test screening (nucleic acid tests, antibody tests, and CT scans) program was required before surgery. If the tests were negative, they could be operated in normal surgery rooms and sent to the gynecologic buffering rooms thereafter. For those who had positive tests and who needed extremely urgent surgery without opportunities to complete preoperative COVID‐19 screening tests, emergency surgery was conducted in specific negative‐pressure surgery rooms followed by an immediate transfer to the isolation wards, where they were treated as suspected COVID‐19 patients. In the isolation wards, the first COVID‐19 triple‐test screening was completed, and patients for whom COVID‐19 was excluded were transferred to the buffering rooms in the gynecologic normal wards (Figure 1). In case of confirmed infection, patients were transferred to designated hospitals for further treatment, and the surgeons who performed the emergency surgery and medical staff in operation rooms were informed.

### Resumption performance

Under the triage framework described above, we received 19 298 patients visits, admitted 326 patients, and performed 223 operations in the first 2 months after the lockdown was lifted (April and May 2020), less than 40% of that in the 2 months before the lockdown (October and November 2020) and the same period in 2019 (April and May 2019; Table 2). A total of 20 121 people, including patients and their caregivers and family members, received ambulatory COVID‐19 screening, 95 patients underwent emergency surgery before completing COVID‐19 screening, and 46 were transferred to designated hospitals because of confirmed or suspected infection. The 46 suspected patients undergo a 14‐day medical observation in the isolation wards for daily body temperature and respiratory symptom monitoring and repeated nucleic acid and serum antibody test weekly. Fortunately, none of them were confirmed.

**TABLE 2:** Characteristics of inpatients before and after COVID‐19 lockdown

	Apr–May 2020	Oct–Nov 2019		Apr–May 2019	
	*N* (%)	*N* (%)	*p*	*N* (%)	*p*
Inpatients					
Number of patients	326	834		844	
Age, year (median, range)	48.5 (14–86)	47.0 (13–84)	0.012^a^	47.0 (14–79)	0.024^a^
Hospital stay, day (median, range)	10.0 (1–29)	9.0 (1–51)	<0.001^a^	9.0 (1–14)	<0.001^a^
Cancer patients	191 (58.6)	417 (50.0)	0.008^b^	372 (44.1)	<0.001^b^
Surgery					
Number of patients	223	622		606	
Age, year (median, range)	46.0 (14–84)	45.0 (17–80)	0.107^a^	45.0 (16–79)	0.180^a^
Cancer patients	101 (45.3)	244 (39.2)	0.114^b^	194 (32.0)	<0.001^b^
Comorbidities					
Severe anemia	4 (1.2)	1 (0.1)	0.010^b^	2 (0.2)	0.034^b^
Hypertension	36 (11.0)	80 (9.6)	0.459^b^	88 (10.4)	0.759^b^
Diabetes mellitus	11 (3.4)	37 (4.4)	0.414^b^	38 (4.5)	0.388^b^
Cancer patients					
Cervical cancer	42 (22.0)	159 (38.1)	<0.001^b^	123 (33.1)	<0.001^b^
Endometrial cancer	30 (15.7)	69 (16.5)		90 (24.2)	
Ovarian cancer	97 (50.8)	168 (40.3)		137 (36.8)	
Others	22 (11.5)	21 (5.0)		22 (5.9)	

*Note: p*, compared to the April–May 2020 cohort.

^a^ Mann–Whitney *U* test.

^b^ Chi‐square test.

In the early phase of the resumption, cancer patients required more effort, especially those suffering from cancers that progress rapidly, such as ovarian cancer. The proportion of cancer patients in April and May 2020 (58.6%) was increased compared to the prelockdown period (October and November 2020, 50.0%) and the same period in 2019 (April and May 2019, 44.1%). Additionally, the percentage of cancer surgery was increased. Among the cancer patients, ovarian cancer became the most frequent disease, while the proportions of cervical cancer and endometrial cancer were decreased compared to the pre‐COVID‐19 periods (Table 2). The ovarian cancer patients with chemotherapy showed less myelosuppression (neutrophil count less than 1.5 G/L) in the postlockdown cohort (16/47, 34.0%) compared to the prelockdown cohort (52/102, 51.0%) and the 2019 cohort (41/95, 43.2%), which may be due to the more frequent use of long‐term treatment with colony stimulating factor (PEG‐rhG‐CSF). However, there were more patients with leiomyoma or adenomyosis suffering from severe anemia in the postlockdown cohort, which may be due to the postponed diagnosis and surgery and inadequate iron supplementation during the COVID‐19 lockdown (Table 2). For gynecological tumor patients, we did not change the original treatment plan because of the COVID‐19 pandemic.

## Discussion

Many countries are now experiencing the second wave of the COVID‐19 epidemic; the battle against COVID‐19 seems unlikely to end soon. Therefore, preventing the transmission of COVID‐19 and preparing to deal with that second wave are very important.^11^ It is evident that the safety of patients and medical staff is the key to resuming treatment for non‐COVID‐19 gynecologic patients. We found that, in the early stage of the resumption of treatment, the medical demands of gynecologic patients who were locked down during the COVID‐19 lockdown increased gradually rather than abruptly after lockdown, which may be partially due to the persistent concerns among patients about exposure to contagions in the hospital. However, in the gynecologic department, the proportion of patients with cancer or leiomyoma and adenomyosis with severe anemia increased, their immunity is often compromised and they are more predisposed to SARS‐CoV‐2 infection.^8^ To prevent cross‐infection in hospital, an effective triage system for non‐COVID‐19 gynecologic patients is very important.

In the present study, we illustrated the workflow that we used to triage gynecologic patients with or without a need for emergency surgery regarding potential COVID‐19. In our triage system, the hospital isolation wards and buffering rooms in the gynecologic department were critical to cut off the transmission pathways of COVID‐19 without delaying the required medical care for patients. Given the possible missed COVID‐19 diagnosis in the primary screening conducted in the outpatient clinic or isolation wards due to infection window periods and false‐negative test results,^12^ the stay in buffering rooms and the second round of screening tests were necessary to protect transmission as a secondary line of defense.

In the triage system we used, the screening tests included nucleic acid testing, chest CT scan, and serologic testing for viral antibodies, which can be completed within 6 h. Although the triage process costs medical resources and increases management burden, it minimizes the risk of nosocomial infections and reassures both patients and healthcare workers, laying the cornerstone for the resumption of medical treatment for non‐COVID‐19 patients. However, the use of this screening regimen demands adequate test capacity and relatively quick performance and might be unfeasible for settings with limited resources. In such cases, a simplified screening regimen with any available nucleic acid test and/or serum antibody test could be considered.^13^ Considering the increased proportion of asymptomatic SARS‐CoV‐2 infection in the postepidemic phase and the relatively low specificity of CT, especially in seasons with high influenza prevalence, chest CT scan can be omitted in the triage process.^14^ To further reduce the testing burden in lower resourced situations, a primary triage with antibody test alone can be considered; an acid test can be used to further triage people with positive antibodies.

The Wuhan Health Committee organized the nucleic acid test of COVID‐19 for nearly 10 million residents from May 14th to June 1st in Wuhan, and only 300 (0.003%) asymptomatic infected individuals were eventually detected. The asymptomatic infected individuals and close contacts have been placed under medical quarantine and no cases of asymptomatic infected individuals transmitted to others. After the comprehensive COVID‐19 screening for permanent residents in Wuhan, the triage system in clinic, inpatient COVID‐19 screening tests, and PPE of medical staff were all simplified in Wuhan hospitals. On December 24th, The Wuhan Health Committee organized the emergency vaccination of COVID‐19 vaccine for medical staff. As COVID‐19 vaccination rates increasing, this workflow was further simplified, such as the isolation wards were canceled and nonemergency patients could be hospitalized with nucleic acid results within 2 weeks. With the group protection immunity gradually forming, this workflow will be released eventually. Given that the COVID‐19 pandemic is a rapidly evolving situation in temporal and spatial contexts, and the conditions within local healthcare systems can be disparate, there is no universal guideline appropriate for all. We provide a model of an effective triage system to avoid transmission and at the same time allow timely treatment for non‐COVID‐19 patients, which may be a meaningful in a setting with relatively sufficient test capacity and medical resources.

## Author Contributions

Liqiong Cai contributes to concept design. Liqiong Cai, Rui Gao and Weihong Dong are major contributors in writing the manuscript. Jing Cai, Shouhua Yang, and Jianfeng Guo collected and interpreted the clinical data. Jing Zhao and Zehua Wang performed the statistical analysis. All authors read and approved the final manuscript.

## Conflict of Interest

The authors declared no potential conflicts of interest.

## Data Availability

The data that support the findings of this study are available from the corresponding author upon reasonable request.
